# Response to “Letter to the Editor Regarding Vaping.” Seven Reasons E-cigarettes Must Be “Downright Prohibited” Now!

**DOI:** 10.5041/RMMJ.10381

**Published:** 2019-10-29

**Authors:** Sharon Galper Grossman

**Affiliations:** Halakhic Advisor (Morah L’Halakha), Matan HaSharon, Ra’annana, Israel; and Oncology Consultant, Ra’annana, Israel

To the Editor,

I thank Rabbi Spitz for his thoughtful analysis. However, I humbly disagree with his conclusion that it is premature to classify e-cigarettes as “downright prohibited.”

His assumption that the *halakhic* process for evaluating the permissibility of e-cigarettes must mirror that for combustible cigarettes is flawed. The known facts 50 years ago, when the Surgeon General issued his report on the dangers of combustible cigarettes, did not give *poskim* reason to prohibit cigarettes immediately. They hesitated because smoking was deeply entrenched in society, confirming that people were willing to accept its dangers and that smoking therefore fell under the rubric of *shomer peta’im Hashem*. Medical data regarding the dangers of smoking unfolded slowly, gradually gaining widespread acceptance. Facing pressure from powerful tobacco lobbies, local and national governments were slow to react and ban use and sales. In addition, it was not clear that society could adhere to a rabbinic prohibition against smoking. *Poskim* mirrored this pace, developing their positions on combustible cigarettes gradually, and only classifying them as *assur* years after data regarding the dangers of smoking were available and once evidence showed that smokers could quit. Sadly, many died during these intervening years.

The e-cigarette story is vastly different. The product is relatively new, without widespread acceptance. Furthermore, it so closely resembles combustible cigarettes, which virtually all *poskim* prohibit, that one could apply the *halakhic* presumption that e-cigarettes are *assur* until medical data confirm without a shadow of doubt that they are substantially safer than combustible cigarettes. The six factors that rendered these products “downright prohibited” at the time of publication of my article are even stronger today, just months later, as the scientific evidence of their danger mounts.

First, while there are no long-term safety data, the limited data in existence point only to the dangers of these products. Since the publication of my article, additional data have provided further evidence that e-cigarettes significantly increase the risk of myocardial infarction, stroke, depression, anxiety, emotional problems,^1^ and impair fertility.^2^ On August 23, 2019, after the US Centers for Disease Control and Prevention (CDC) reported 193 cases of severe respiratory illnesses after vaping, including one death, health officials declared: “We must get the word out that using e-cigarettes and vaping is dangerous.”^3^ The close resemblance of these products to combustible cigarettes, which are prohibited, and the growing evidence of their long-term dangers render them at the least *safek sakana*, which the Rama prohibits, and perhaps even a *vaday sakana*, which is certainly prohibited.

Second, there is mounting evidence that society is not willing to accept the dangers of these products, thus removing them from the protection of *shomer peta’im Hashem*. Tsitz Eliezer cites warning labels and government action against combustible cigarettes as evidence that combustible cigarettes no longer fall under *shomer peta’im Hashem*. Although the Surgeon General’s warnings regarding e-cigarettes have been tepid, his position and that of the US Food and Drug administration (FDA), CDC, and virtually all relevant national medical organizations on the dangers of these products in youth and pregnant women are unequivocal. A Joint Statement issued on August 30, 2019 by the CDC and FDA announced: “Regardless of the ongoing investigation [into the current vaping-related deaths], e-cigarette products should not be used by youth, young adults, pregnant women, as well as adults who do not currently use tobacco products.”^4^ Is it premature to declare e-cigarette use “downright prohibited” for these populations? Various local governments and states have restricted e-cigarettes, providing further evidence that society does not accept the risks of these products. San Francisco banned sales of e-cigarettes,^5^ and North Carolina filed suit against JUUL for “misrepresenting the potency and danger of nicotine in its products.”^6^ In contrast, despite years of evidence of the dangers of combustible cigarettes, only one US city—Beverly Hills—banned their sale (and that only this past June).^7^ This legal action against e-cigarettes moves them from *shomer peta’im Hashem* to prohibited.

Third, even if one argues that the data are insufficient to prove that e-cigarettes are dangerous or that society is unwilling to accept these dangers, the reality that these products are a gateway to combustible cigarettes and a lifetime of cigarette smoking—which almost all *poskim* prohibit—renders them *assur* as a means to sin. In 2011, Rav Ovadia Yosef decried the dangers of smoking, declaring: “Whoever can refrain from it [smoking], all the better; he should take *every effort* to keep away from it.”^8^ Similarly, Shevet HaLevi confirmed the importance of keeping people from starting smoking. As e-cigarettes are a gateway to combustible cigarettes, these calls to action should include prohibiting e-cigarettes.

Fourth, e-cigarettes lead to nicotine as well as opioid addiction, which Rav Moshe Feinstein clearly prohibits.

Fifth, e-cigarettes expose bystanders to second-hand smoke, which the Surgeon General has declared harmful. While some *poskim* forbid second-hand smoke exposure only when bystanders find it offensive, Shevet HaLevi categorically prohibits smoking in public places, regardless of whether people were complaining that it bothered them.^9^

Sixth, the *poskim* with whom I have spoken have *unambiguously and absolutely* proscribed e-cigarette use. In *Kashrus* magazine, Rav Sternbuch does not address the permissibility of e-cigarettes or whether they fall under *shomer peta’im Hashem* but rather the question of whether they require kosher certification. Because the flavorings provide *hana’ah*, gratification, he concludes that they must have it. This requirement and his omission of any prohibition against these products themselves are not “implicit proof” of their permissibility but, rather, a recognition of the reality that the flavors are enjoyable, a reality that has increased the popularity of these products, especially for first-time users who might otherwise have found the taste of these products unpleasant. Any conclusions regarding his position on e-cigarette use is purely speculative.

However, the nail in the coffin for vaping is best reflected by the most recent publication of Layden et al. in the *New England Journal of Medicine* on September 6, 2019 which reported on a series of severe pulmonary disease cases associated with e-cigarette usage.^10^ Commenting on this and other findings, Dr David C. Christiani wrote in an editorial in that same issue: “physicians should discourage their patients from vaping,” even though the precise connection between vaping and these newly described pulmonary illnesses has not been thoroughly investigated.^11^ Hence, if physicians are now seeking to prevent vaping—despite their lack of knowledge on precise correlation with death and illness—the *halakhic* stand should be just as strong, if not more so.

The current facts justify prohibiting e-cigarettes now; to delay just because *poskim* waited many years before prohibiting combustible cigarettes does not reinforce the *halakhic* process but rather ignores and undermines it. Analysis of the permissibility of e-cigarettes begins with and builds upon *halakha*’s nearly unequivocal denunciation of smoking and anything that resembles or leads to it. To hesitate is to disregard these *halakhic* rulings. How many lives could have been saved had the social realities of combustible cigarettes been similar to those of e-cigarettes?

In a recent congressional hearing investigating the dangers of JUUL, Congresswoman Ayana Presley stated: “I would be remiss not to highlight how similar many of JUUL’s tactics seem to be right out of the Big Tobacco playbook. ... It’s extremely disturbing, we’ve been here before. We don’t need a bunch of studies. The only studies we need are the millions of casualties that are behind us and that we run the risk of seeing ahead of us.”^12^ We have been here before. We must build on the lessons we have learned from combustible cigarettes and do everything in our power to inform the public of the dangers and *halakhic* prohibitions of e-cigarettes in order to prevent unnecessary loss of human life. May the example of the Chofetz Chaim, who prohibited combustible cigarettes nearly 50 years before the Surgeon General’s landmark report, serve as a model for how *halakha* approaches e-cigarettes. How many more deaths must occur before we declare e-cigarettes “downright prohibited”?

## Abbreviations

CDC: US Centers for Disease Control and Prevention
FDA: US Food and Drug Administration.
